# Butan-1-aminium tetra­chlorido­ferrate(III)–18-crown-6 (1/1)

**DOI:** 10.1107/S160053681204994X

**Published:** 2012-12-12

**Authors:** Yuan Zhang

**Affiliations:** a Department of Applied Chemistry, Nanjing College of Chemical Technology, Nanjing 210048, People’s Republic of China

## Abstract

In the crystal of the title compound, (C_4_H_12_N)[FeCl_4_]·C_12_H_24_O_6_, the butan-1-aminium cation and the tetra­chloridoferrate(III) anion have *m* symmetry: in the cation, the non-H atoms are located on the mirror plane and in the anion, the Fe^III^ atom and two Cl atoms are located on the mirror plane. The 18-crown-6 mol­ecule also has *m* symmetry, with two O atoms located on the mirror plane. The butan-1-amine cation and the 18-crown-6 mol­ecule are connected by N—H⋯O hydrogen bonds.

## Related literature

For related co-crystals of (18-crown-6)] and anilinium salts, see: Akutagawa *et al.* (2009 ▶).
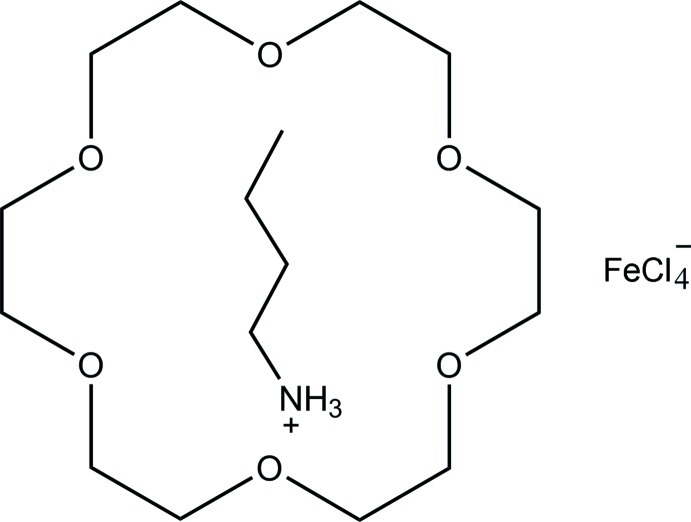


## Experimental

### 

#### Crystal data

(C_4_H_12_N)[FeCl_4_]·C_12_H_24_O_6_
*M* *_r_* = 536.11
Orthorhombic, 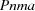

*a* = 9.3109 (19) Å
*b* = 11.431 (2) Å
*c* = 24.718 (5) Å
*V* = 2630.8 (9) Å^3^
*Z* = 4
Mo *K*α radiation
μ = 1.01 mm^−1^
*T* = 293 K
0.40 × 0.30 × 0.20 mm

#### Data collection

Rigaku SCXmini diffractometer
Absorption correction: multi-scan (*CrystalClear*; Rigaku, 2005 ▶) *T*_min_ = 0.665, *T*_max_ = 0.820
24682 measured reflections
3160 independent reflections
1649 reflections with *I* > 2σ(*I*)
*R*_int_ = 0.090

#### Refinement

*R*[*F* ^2^ > 2σ(*F* ^2^)] = 0.079
*wR*(*F* ^2^) = 0.234
*S* = 1.04
3160 reflections
142 parameters
H-atom parameters constrained
Δρ_max_ = 0.74 e Å^−3^
Δρ_min_ = −0.40 e Å^−3^

### 

Data collection: *CrystalClear* (Rigaku, 2005 ▶); cell refinement: *CrystalClear*; data reduction: *CrystalClear*; program(s) used to solve structure: *SHELXS97* (Sheldrick, 2008 ▶); program(s) used to refine structure: *SHELXL97* (Sheldrick, 2008 ▶); molecular graphics: *SHELXTL* (Sheldrick, 2008 ▶); software used to prepare material for publication: *SHELXTL*.

## Supplementary Material

Crystal structure: contains datablock(s) I, global. DOI: 10.1107/S160053681204994X/xu5660sup1.cif

Structure factors: contains datablock(s) I. DOI: 10.1107/S160053681204994X/xu5660Isup2.hkl

Additional supplementary materials:  crystallographic information; 3D view; checkCIF report

## Figures and Tables

**Table 1:** Hydrogen-bond geometry (Å, °)

*D*—H⋯*A*	*D*—H	H⋯*A*	*D*⋯*A*	*D*—H⋯*A*
N1—H1*A*⋯O1	0.90	2.07	2.963 (5)	176
N1—H1*B*⋯O3	0.90	2.07	2.966 (4)	175

**Fig. 1.**
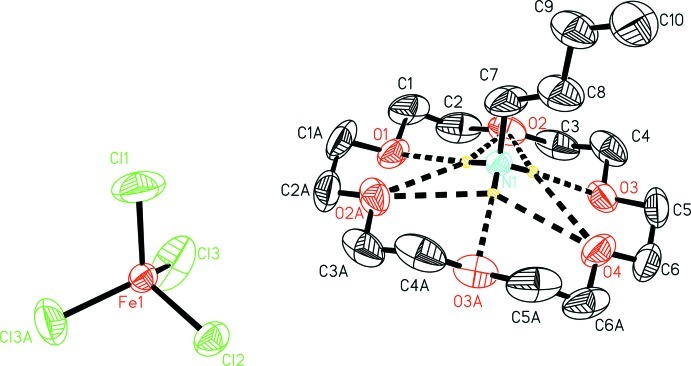
Perspective structure of the title compound, showing the atom-numbering scheme. Displacement ellipsoids are drawn at the 30% probability level.

## supplementary crystallographic information

### Comment

Several supramolecular rotators of [(Ani)(18-crown-6)]^+^ and [(Ani)(dibenzo18-crown-6)]^+^ in [Ni(dmit)_2_]^-^ salts (dmit_2_^-^ = 2-thioxo-1,3,dithiole-4,5-dithiolate; Ani^+^ = anilinium) have been reported, of which some display novel ferroelectric features (Akutagawa *et al.*, 2009). There is a class of transition metal complexes whose electronic and magnetic properties in the solid state arise from the extended π-ligands (π-electrons and π-spins). So we try to replace the amine, crown ether and [Ni(dmit)_2_]^-^ salts. Herein, we report the [tetrachloro-iron-anion] which replace the [Ni(dmit)_2_]^-^ salts providing high electrical conductivity.

Supramolecular rotators was assembled between protonated butan-1-amine (C_4_H_9_NH_3_)^+^and 18-crown-6 by of hydrogen-bonding. The ammonium moieties of (–NH3+) cations were interacted with the six oxygen atoms of crown ethers through six simple N–H···O hydrogen bonding, forming 1:1 (one crown ether ring per ammonium group) supramolecular rotator-stator structures. The FeCl_4_^-^ anions are relatively small for embedding large and structurally diverse supramolecular cations in the crystal lattice. Again, such a feature further supports the fact that the size and shape of the supramolecular assemblies between C_4_H_9_NH_3_^+^ and crown ethers are strongly affected by the proton-transfer state.

### Experimental

C_4_H_9_NH_2_.HCl(4 mmol, 0.442 g) and 18-crown-6 (4 mmol, 1.056 g) were dissolved in methanol solution. After addition of tervalent ferric chloride (4 mmol, 1.08 g) in concentrated hydrochloric acid medium, the precipitate was filtered and washed with a small amount of methanol. Single crystals suitable for X-ray diffraction analysis were obtained from slow evaporation of methanol and DMF solution at room temperature after two days.

### Refinement

H atoms were positioned geometrically and refined using a riding model, with C—H = 0.97 Å and *U*_iso_(H) = 1.2_eq_(C).

### Crystal data

**Table:**

(C_4_H_12_N)[FeCl_4_]·C_12_H_24_O_6_	*F*(000) = 1124
*M**_r_* = 536.11	*D*_x_ = 1.354 Mg m^−^^3^
Orthorhombic, *P**n**m**a*	Mo *K*α radiation, λ = 0.71073 Å
Hall symbol: -P 2ac 2n	Cell parameters from 3160 reflections
*a* = 9.3109 (19) Å	θ = 2.4–27.5°
*b* = 11.431 (2) Å	µ = 1.01 mm^−^^1^
*c* = 24.718 (5) Å	*T* = 293 K
*V* = 2630.8 (9) Å^3^	Prism, colorless
*Z* = 4	0.40 × 0.30 × 0.20 mm

### Data collection

**Table:**

Rigaku SCXmini diffractometer	3160 independent reflections
Radiation source: fine-focus sealed tube	1649 reflections with *I* > 2σ(*I*)
Graphite monochromator	*R*_int_ = 0.090
Detector resolution: 13.6612 pixels mm^-1^	θ_max_ = 27.5°, θ_min_ = 3.1°
CCD_Profile_fitting scans	*h* = −12→12
Absorption correction: multi-scan (*CrystalClear*; Rigaku, 2005)	*k* = −14→14
*T*_min_ = 0.665, *T*_max_ = 0.820	*l* = −32→32
24682 measured reflections	

### Refinement

**Table:**

Refinement on *F*^2^	Primary atom site location: structure-invariant direct methods
Least-squares matrix: full	Secondary atom site location: difference Fourier map
*R*[*F*^2^ > 2σ(*F*^2^)] = 0.079	Hydrogen site location: inferred from neighbouring sites
*wR*(*F*^2^) = 0.234	H-atom parameters constrained
*S* = 1.04	*w* = 1/[σ^2^(*F*_o_^2^) + (0.0881*P*)^2^ + 3.1745*P*] where *P* = (*F*_o_^2^ + 2*F*_c_^2^)/3
3160 reflections	(Δ/σ)_max_ = 0.001
142 parameters	Δρ_max_ = 0.74 e Å^−^^3^
0 restraints	Δρ_min_ = −0.40 e Å^−^^3^

### Special details

**Table:**

Geometry. All esds (except the esd in the dihedral angle between two l.s. planes) are estimated using the full covariance matrix. The cell esds are taken into account individually in the estimation of esds in distances, angles and torsion angles; correlations between esds in cell parameters are only used when they are defined by crystal symmetry. An approximate (isotropic) treatment of cell esds is used for estimating esds involving l.s. planes.
Refinement. Refinement of F^2^ against ALL reflections. The weighted R-factor wR and goodness of fit S are based on F^2^, conventional R-factors R are based on F, with F set to zero for negative F^2^. The threshold expression of F^2^ > 2sigma(F^2^) is used only for calculating R-factors(gt) etc. and is not relevant to the choice of reflections for refinement. R-factors based on F^2^ are statistically about twice as large as those based on F, and R- factors based on ALL data will be even larger.

### Fractional atomic coordinates and isotropic or equivalent isotropic displacement parameters (Å^2^)

**Table:**

	*x*	*y*	*z*	*U*_iso_*/*U*_eq_	
Fe1	0.22073 (9)	0.2500	0.88238 (3)	0.0713 (3)	
Cl1	0.4469 (2)	0.2500	0.86376 (15)	0.1792 (13)	
Cl2	0.0976 (2)	0.2500	0.80773 (6)	0.0961 (6)	
Cl3	0.1710 (3)	0.40675 (17)	0.92687 (8)	0.1835 (8)	
N1	0.3475 (4)	0.7500	0.65867 (15)	0.0562 (11)	
H1A	0.3430	0.7500	0.6950	0.084*	
H1B	0.3034	0.8143	0.6457	0.084*	
C8	0.5254 (9)	0.7500	0.5846 (4)	0.133 (3)	
H8A	0.4780	0.6824	0.5700	0.159*	
C9	0.6662 (8)	0.7500	0.5629 (4)	0.129 (3)	
H9A	0.7139	0.6822	0.5771	0.155*	
C7	0.4966 (7)	0.7500	0.6410 (3)	0.131 (4)	
H7A	0.5430	0.6822	0.6559	0.157*	
C10	0.6838 (10)	0.7500	0.5070 (4)	0.175 (5)	
H10A	0.7837	0.7500	0.4973	0.262*	
H10B	0.6384	0.6814	0.4925	0.262*	
O4	0.1585 (5)	0.7500	0.56513 (17)	0.0986 (16)	
C6	0.0868 (5)	0.8539 (6)	0.54992 (19)	0.121 (2)	
H6A	−0.0039	0.8575	0.5683	0.145*	
H6B	0.0687	0.8537	0.5117	0.145*	
O1	0.3486 (5)	0.7500	0.77856 (16)	0.0929 (15)	
C1	0.4203 (6)	0.8546 (6)	0.79449 (19)	0.127 (2)	
H1C	0.5134	0.8572	0.7778	0.152*	
H1D	0.4331	0.8557	0.8330	0.152*	
O2	0.3379 (3)	0.9612 (3)	0.71993 (14)	0.0964 (10)	
O3	0.1868 (3)	0.9589 (3)	0.62119 (14)	0.0938 (10)	
C4	0.2686 (6)	1.0561 (5)	0.6389 (3)	0.121 (2)	
H4A	0.2304	1.1272	0.6240	0.145*	
H4B	0.3662	1.0480	0.6270	0.145*	
C5	0.1737 (6)	0.9561 (6)	0.5645 (2)	0.127 (2)	
H5A	0.1275	1.0264	0.5524	0.153*	
H5B	0.2662	0.9511	0.5475	0.153*	
C3	0.2649 (6)	1.0613 (4)	0.6980 (3)	0.121 (2)	
H3A	0.3112	1.1312	0.7106	0.145*	
H3B	0.1668	1.0626	0.7100	0.145*	
C2	0.3375 (6)	0.9578 (6)	0.7762 (2)	0.129 (2)	
H2A	0.3783	1.0284	0.7906	0.154*	
H2B	0.2407	0.9508	0.7891	0.154*	

### Atomic displacement parameters (Å^2^)

**Table:**

	*U*^11^	*U*^22^	*U*^33^	*U*^12^	*U*^13^	*U*^23^
Fe1	0.0715 (5)	0.0665 (5)	0.0757 (5)	0.000	−0.0136 (4)	0.000
Cl1	0.0628 (12)	0.189 (3)	0.285 (4)	0.000	−0.0184 (17)	0.000
Cl2	0.0920 (11)	0.1220 (14)	0.0744 (9)	0.000	−0.0151 (9)	0.000
Cl3	0.256 (2)	0.1460 (13)	0.1486 (13)	0.0826 (14)	−0.0819 (13)	−0.0772 (11)
N1	0.048 (2)	0.068 (3)	0.053 (2)	0.000	0.0015 (19)	0.000
C8	0.092 (5)	0.148 (8)	0.158 (8)	0.000	0.039 (5)	0.000
C9	0.096 (5)	0.128 (7)	0.163 (8)	0.000	0.046 (5)	0.000
C7	0.059 (4)	0.239 (11)	0.096 (5)	0.000	0.013 (4)	0.000
C10	0.132 (7)	0.300 (16)	0.093 (6)	0.000	0.035 (6)	0.000
O4	0.075 (3)	0.156 (4)	0.065 (2)	0.000	−0.014 (2)	0.000
C6	0.080 (3)	0.208 (6)	0.075 (3)	0.026 (4)	−0.004 (2)	0.049 (4)
O1	0.075 (3)	0.142 (4)	0.061 (2)	0.000	−0.016 (2)	0.000
C1	0.103 (4)	0.205 (6)	0.072 (3)	−0.041 (4)	−0.021 (3)	−0.037 (4)
O2	0.095 (2)	0.078 (2)	0.116 (2)	−0.0204 (17)	0.0195 (18)	−0.0302 (18)
O3	0.0780 (19)	0.082 (2)	0.121 (2)	0.0047 (16)	0.0201 (17)	0.0362 (18)
C4	0.105 (4)	0.067 (3)	0.190 (6)	0.014 (3)	0.047 (4)	0.034 (3)
C5	0.100 (4)	0.182 (5)	0.100 (3)	0.035 (4)	0.017 (3)	0.083 (4)
C3	0.094 (3)	0.051 (3)	0.217 (7)	−0.011 (3)	0.037 (4)	−0.023 (4)
C2	0.111 (4)	0.148 (5)	0.127 (4)	−0.039 (4)	−0.001 (3)	−0.072 (4)

### Geometric parameters (Å, º)

**Table:**

Fe1—Cl3	2.1528 (18)	C6—H6B	0.9599
Fe1—Cl3^i^	2.1528 (18)	O1—C1	1.425 (6)
Fe1—Cl1	2.155 (2)	O1—C1^ii^	1.425 (6)
Fe1—Cl2	2.1723 (18)	C1—C2	1.480 (9)
N1—C7	1.456 (8)	C1—H1C	0.9600
N1—H1A	0.9000	C1—H1D	0.9601
N1—H1B	0.9001	O2—C2	1.391 (6)
C8—C9	1.416 (10)	O2—C3	1.437 (6)
C8—C7	1.419 (10)	O3—C5	1.406 (6)
C8—H8A	0.9600	O3—C4	1.416 (7)
C9—C10	1.392 (12)	C4—C3	1.463 (9)
C9—H9A	0.9600	C4—H4A	0.9601
C7—H7A	0.9600	C4—H4B	0.9600
C10—H10A	0.9601	C5—H5A	0.9600
C10—H10B	0.9601	C5—H5B	0.9600
O4—C6^ii^	1.413 (6)	C3—H3A	0.9599
O4—C6	1.413 (6)	C3—H3B	0.9600
C6—C5	1.466 (9)	C2—H2A	0.9601
C6—H6A	0.9600	C2—H2B	0.9601
			
Cl3—Fe1—Cl3^i^	112.68 (13)	C2—C1—H1C	108.3
Cl3—Fe1—Cl1	108.61 (8)	O1—C1—H1D	110.1
Cl3^i^—Fe1—Cl1	108.61 (8)	C2—C1—H1D	110.9
Cl3—Fe1—Cl2	108.69 (6)	H1C—C1—H1D	108.3
Cl3^i^—Fe1—Cl2	108.69 (6)	C2—O2—C3	113.4 (5)
Cl1—Fe1—Cl2	109.52 (12)	C5—O3—C4	111.9 (4)
C7—N1—H1A	110.2	O3—C4—C3	109.1 (4)
C7—N1—H1B	109.1	O3—C4—H4A	110.3
H1A—N1—H1B	109.5	C3—C4—H4A	109.9
C9—C8—C7	123.1 (8)	O3—C4—H4B	109.8
C9—C8—H8A	106.4	C3—C4—H4B	109.4
C7—C8—H8A	106.5	H4A—C4—H4B	108.3
C10—C9—C8	119.0 (9)	O3—C5—C6	108.2 (4)
C10—C9—H9A	107.9	O3—C5—H5A	109.2
C8—C9—H9A	106.9	C6—C5—H5A	110.0
C8—C7—N1	118.4 (6)	O3—C5—H5B	111.1
C8—C7—H7A	106.9	C6—C5—H5B	109.9
N1—C7—H7A	108.3	H5A—C5—H5B	108.4
C9—C10—H10A	111.2	O2—C3—C4	109.5 (4)
C9—C10—H10B	108.6	O2—C3—H3A	109.2
H10A—C10—H10B	109.5	C4—C3—H3A	110.3
C6^ii^—O4—C6	114.4 (6)	O2—C3—H3B	110.2
O4—C6—C5	110.1 (4)	C4—C3—H3B	109.3
O4—C6—H6A	109.0	H3A—C3—H3B	108.3
C5—C6—H6A	109.6	O2—C2—C1	109.0 (5)
O4—C6—H6B	110.1	O2—C2—H2A	110.3
C5—C6—H6B	109.9	C1—C2—H2A	110.5
H6A—C6—H6B	108.2	O2—C2—H2B	109.7
C1—O1—C1^ii^	114.1 (6)	C1—C2—H2B	108.7
O1—C1—C2	109.9 (4)	H2A—C2—H2B	108.5
O1—C1—H1C	109.3		

Symmetry codes: (i) *x*, −*y*+1/2, *z*; (ii) *x*, −*y*+3/2, *z*.

### Hydrogen-bond geometry (Å, º)

**Table:**

*D*—H···*A*	*D*—H	H···*A*	*D*···*A*	*D*—H···*A*
N1—H1*A*···O1	0.90	2.07	2.963 (5)	176
N1—H1*B*···O3	0.90	2.07	2.966 (4)	175
